# 

**Published:** 1881-06

**Authors:** 


### SPECIAL NOTICE.

EXCHANGES, BOOKS for notice or review, and ALL COMMUNICATIONS appertaining to the editorial department other than dental, *should be*

*sent in future to* Dr. Harvey L. Byrd, *No.* 225 *N. Gilmor street, Baltimore, Md.;* and *all dental* matters should be sent, as heretofore, to Dr. B. M. Wilkerson, *No.* 68 *N. Charles street, Baltimore, Md.*

All matters relating to the *business* management of the Journal should be directed to The Independent Practitioner, *No.* 20 *Cortlandt Street, New York City.*

*We take the privilege of deciding what we shall publish, but the author only is responsible for his production.*

We have facilities which enable us to make liberal discounts to our cash subscribers on text and many other books, also Webster's Unabridged Dictionary, the various publications of Frank Leslie and Harper, Scribner's Monthly, Lippincott's Magazine, North American Review, Popular Science Monthly, &c., &c., also many of the leading daily and weekly news and pictorial papers.

AN EXTRA

INDUCEMENT

*To Subscribers.*

See the Advertisement of the Johnson Revolving Book Case in the May issue.

This column will be sold for advertising space six months during the year at *special rates.*

Money is at OUR RISK ONLY when sent by Post Office Money Order, Registered Letter, or Check on Banks in New York City, made payable to INDEPENDENT PRACTITIONER, 20 Courtlandt street, N. Y. City.

If you prefer to send check on your private bank, unless located in the city

